# Cardiomyopathy Classification: Ongoing Debate in the Genomics Era

**DOI:** 10.1155/2012/796926

**Published:** 2012-08-08

**Authors:** Charles McCartan, Robert Mason, S. R. Jayasinghe, Lyn R. Griffiths

**Affiliations:** ^1^Genomics Research Centre, Griffith Health Institute, Griffith University, Parklands Drive, Southport, QLD 4222, Australia; ^2^Department of Cardiology, Gold Coast Hospital, Southport Campus, Nerang Street, Southport, QLD 4218, Australia

## Abstract

Cardiomyopathies represent a group of diseases of the myocardium of the heart and include diseases both primarily of the cardiac muscle and systemic diseases leading to adverse effects on the heart muscle size, shape, and function. Traditionally cardiomyopathies were defined according to phenotypical appearance. Now, as our understanding of the pathophysiology of the different entities classified under each of the different phenotypes improves and our knowledge of the molecular and genetic basis for these entities progresses, the traditional classifications seem oversimplistic and do not reflect current understanding of this myriad of diseases and disease processes. Although our knowledge of the exact basis of many of the disease processes of cardiomyopathies is still in its infancy, it is important to have a classification system that has the ability to incorporate the coming tide of molecular and genetic information. This paper discusses how the traditional classification of cardiomyopathies based on morphology has evolved due to rapid advances in our understanding of the genetic and molecular basis for many of these clinical entities.

## 1. Introduction

Cardiomyopathies are a broad spectrum of diseases that affect the muscle or myocardium of the heart. This results in a failure of the heart to provide adequate oxygenated blood to the body and remove carbon dioxide and other waste products. The heart is an extremely specialised, richly innervated muscular pump that is designed to beat continuously, without stopping for the entire lifespan of its owner. To put this in perspective, a human heart beating at 70 bpm will beat approximately 2.5 billion times during a 70-year lifespan.

The official definition of cardiomyopathy by the American Heart Association in 2006 is as follows.

*“Cardiomyopathies are a heterogeneous group of diseases of the myocardium associated with mechanical and/or electrical dysfunction that usually (but not invariably) exhibit inappropriate ventricular hypertrophy or dilatation and are due to a variety of causes that frequently are genetic. Cardiomyopathies either are confined to the heart or are part of generalized systemic disorders, which may lead to cardiovascular death or progressive heart failure-related disability [1].”*

## 2. Classification

There are many ways to classify cardiomyopathies. Previously, a cardiomyopathy was defined as “a heart muscle disease of unknown cause” [3] and was broken down according to their pathophysiological phenotype into dilated cardiomyopathy, hypertrophic cardiomyopathy, or restrictive cardiomyopathy. Since this first classification, major advances have meant that this overly simplistic system needed a more indepth approach to incorporate new clinical entities such as arrhythmogenic right ventricular dysplasia (ARVD) [3, 4].

Therefore in 1995, a task force established by the WHO/ISFC compiled a new system which included ARVD and unclassified cardiomyopathies (e.g., fibroelastosis, noncompacted myocardium, systolic dysfunction with minimal dilatation, and mitochondrial involvement). The term “specific cardiomyopathy” was used to describe heart muscle disorders which are as a result of cardiac or systemic diseases such as coronary artery disease, valvular heart disease, or hypertension [61].

A more complete classification based on the AHA definition above divides cardiomyopathies into (1) primary cardiomyopathies, which affect the heart alone, and (2) secondary cardiomyopathies, which are the result of a systemic illness affecting many other parts of the body. These are then further broken down into subgroups within these two broad categories incorporating new genetic and molecular insights (Table 1).

Distinguishing between primary and secondary cardiomyopathies can be challenging as many diseases classified as primary can have extra cardiac components, and many secondary cardiomyopathies can mainly or exclusively affect the heart. Whether or not this is the best method of classification has generated some debate within the literature [62, 63]. With our growing understanding of the genomic and molecular markers made available by modern laboratory research, a new approach had been proposed to address this overlap based on the causative mutation implicated in causing the disease [64, 65]. The working group from the European Society of Cardiology (ESC) in 2008 defined cardiomyopathy as follows.

*“A myocardial disorder in which the heart muscle is structurally and functionally abnormal, in the absence of coronary artery disease, hypertension, valvular disease and congenital heart disease sufficient to cause the observed myocardial abnormality [2].”*

**Figure 1:**
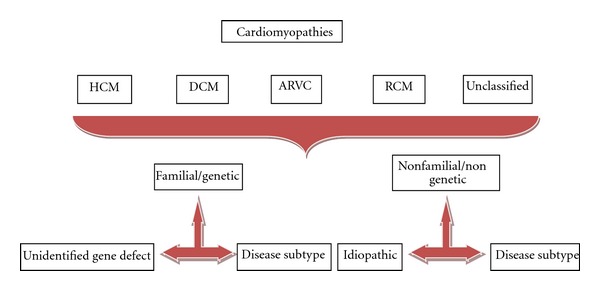
Summary of ESC 2008 Classification [2]. DCM: dilated cardiomyopathy; HCM: hypertrophic cardiomyopathy; ARVC: arrhythmogenic right ventricular cardiomyopathy; RCM: restrictive cardiomyopathy.

While diagnosis of cardiomyopathy rarely begins with the identification of a genetic mutation, it is rational to incorporate genetic mutation testing within a framework of classification as it is important while formulating a plan of treatment and also in informing families of their prognosis (Figure 1).

### 2.1. Hypertrophic Cardiomyopathy (HCM)

Hypertrophic cardiomyopathy (HCM) has been defined by the presence of myocardial hypertrophy incongruent with the haemodynamic stress required for the degree of hypertrophy and the exclusion of infiltrative diseases such as amyloidosis and storage diseases [2, 66, 67].

In the absence of hypertension and valve disease, left ventricular hypertrophy (LVH) occurs in approximately 1 : 500 of the general population [68]. In day-to-day clinical practice it is very difficult to differentiate between pathologies using minimally invasive techniques such as cardiac echo or cardiac magnetic resonance imaging (MRI). Histological demonstration (on myocardial biopsy) of myocyte hypertrophy in the definition of HCM is unreliable due to the patchy nature of the abnormality within the myocardium. The position statement from the ESC [2] contained the following “the presence of intramyocardial storage material is not an exclusion criterion for HCM…. Instead, hypertrophic cardiomyopathies are simply defined by the presence of increased ventricular wall thickness or mass in the absence of loading conditions (hypertension, valve disease) sufficient to cause the observed abnormality.” The “potential inaccuracy” in not fully excluding infiltrative disease or demonstrating myocyte hypertrophy on biopsy is justified by leading to increased emphasis in the clinical picture and a promise of better minimally invasive diagnostic strategies.

If the HCM is familial, then it is usually transmitted in an autosomal dominant pattern of inheritance caused by mutations within genes that encode for various proteins of the cardiac sarcomere. Currently, there are over 500 mutations in 13 genes that have been identified that cause HCM and 50% of these are familial [66, 69–72] (Table 2).

Pathologically, left ventricular (LV) cavity size is normally reduced and this can progress to LV dilatation and heart failure, albeit in a minority of patients. There are many patterns of hypertrophy and all are consistent with a diagnosis of HCM but concentric hypertrophy is more suggestive of a systemic cause such as glycogen storage disease. Moreover, mutations in the genes encoding for cardiac troponins can be associated with mild phenotypes but, conversely, a high incidence of cardiac death [73]. The normal physiological hypertrophy that occurs in highly competitive athletes is uncommon (less than 2% of male athletes) [74], but it is important not to miss HCM in these individuals as the risk of sudden death is unacceptably high [75] and causes great distress to both families and communities who have been affected.

### 2.2. Restrictive Cardiomyopathy

Restrictive cardiomyopathies have a diverse range of aetiology; however, all are recognised as having distinct haemodynamic features separating them from other forms of cardiomyopathy. Restrictive cardiomyopathies in general are defined as showing normal ventricular size (nondilated and nonhypertrophied) with impaired haemodynamic function, elevated filling pressures, and diastolic dysfunction, and in most cases normal systolic function [76, 77].

Presentation can include symptoms of both right and left sided failure; decreased exercise tolerance, dyspnoea, peripheral oedema, and palpitations are the most common symptoms [77]. Due to the contrast in both aetiology and treatment options and the similarities in haemodynamics, it is important to recognise the difference between restrictive cardiomyopathy and constrictive pericarditis. Usually, this is defined with a variety of investigatory modalities with both haemodynamic and morphological assessment and includes echocardiography and pericardial imaging [78].

Various aetiologies have been identified as causing restrictive cardiomyopathy and range from idiopathic (primary) restrictive cardiomyopathy, to systemic conditions including infiltrative, noninfiltrative, and storage disorders, as well as endomyocardial disorders, various medications, and iatrogenic causes [79]. Familial restrictive cardiomyopathies are usually inherited in an autosomal dominant fashion, the genetic basis of which remains to be identified, and are noted to be relatively rare [80]. Hereditary conditions known to cause a restrictive cardiomyopathy include haemochromatosis, glycogen storage diseases, Fabry's disease, Gaucher's disease, and Hurler syndrome.

Prognosis in symptomatic patients is quite poor, depending on aetiology. Idiopathic restrictive cardiomyopathy has been associated with a significant difference in 10-yr survival when compared to expected survival in groups matched for age and sex [81]. In comparison with other forms of cardiomyopathy, restrictive cardiomyopathy is relatively uncommon, though it still demonstrates an appreciable incidence in some population groups, namely, Asia, South and Central America [79].

### 2.3. Dilated Cardiomyopathy (DCM)

DCM is a common cause of congestive cardiac failure (CCF) and is defined by the presence left ventricular systolic dysfunction with left ventricular dilatation the absence of coronary artery disease or other causes such as hypertension or valvular pathology [2]. The right ventricle may be involved but is not necessary for the diagnosis. The exact prevalence of DCM in the general population is unknown, but it clearly varies with age and geography and is the most common diagnosis in patients referred for cardiac transplantation [82, 83]. Around 30–50% of cases have a familial component [71, 84], and more than 30 genes have been identified, to date, that cause DCM (Table 3). Most are inherited in an autosomal dominant fashion although some can be autosomal recessive, X-linked or mitochondrial. The actual frequency of familial DCM is probably underestimated.

The 2009 HFSA [71] has released guidelines on the diagnosis and treatment of patients with DCM. A careful family history of three or more generations of family members should be elicited including unexplained heart failure and sudden death in family members before the age of 60 without any symptoms of coronary artery disease. The diagnosis of familial DCM can be made when there are three or more close family members with unexplained DCM. Screening of family members can then take place; this should happen with or without genetic testing and is supported by the fact that many patients can be asymptomatic despite being affected. The 2009 HFSA made the following recommendations for screening: full history, focusing on symptoms of heart failure (dyspnoea, syncope, presyncope, and palpitations); physical examination; ECG; Echo; CK MM. First-degree relatives who have negative findings on initial screening should be rescreened in three- to five-year intervals, but if there are any abnormal findings during the initial screen, the patient should be rescreened in one year.

Peripartum cardiomyopathy is a specific subgroup of dilated cardiomyopathy defined as the development of heart failure with evidence of left ventricular dysfunction, within the last month of pregnancy to within 5 months of delivery, without other identifiable cause or underlying cardiac condition [85, 86]. Groups of women presenting during the earlier stages of pregnancy have been identified and with similar epidemiological characteristics and with similar disease progression and outcomes. The earlier time frame of presentation has been postulated to represent part of a spectrum of peripartum cardiomyopathy [87]. In the group of women presenting in the early stages of pregnancy, search for underlying cardiac conditions (valvular, ischaemic, and myocardial) should be approached. Peripartum cardiomyopathy affects approximately 1 : 4000 women across the US and Europe each year, with higher rates noted across the African continent [88]. The condition usually presents with dyspnoea, cough, peripheral oedema, orthopnoea, paroxysmal nocturnal dyspnoea, generalised fatigue, and chest discomfort. Investigations including new ECG finding of arrhythmia, chest X-ray with foetal shielding (if required for diagnosis of pulmonary oedema) showing cardiomegaly, pulmonary venous congestion and interstitial oedema, and an elevated BNP or NT-proBNP level further suggest the presence of peripartum cardiomyopathy [89]. Echocardiographic evidence demonstrating LV enlargement, a LV end-systolic dimension greater than 2.7 cm/m² of body surface area, LVEF less than 45% and/or fractional shortening less than 30 percent, conclude the presence of heart failure. The use of cardiac MRI in the diagnosis and evaluation of peripartum cardiomyopathy is currently being explored and the presence or lack thereof of late gadolinium enhancement as a prognostic feature in peripartum cardiomyopathy [90, 91]. The aetiology of peripartum cardiomyopathy has been unclear for many years; however, new research into an inflammatory or immunological basis, and the role of prolactin in the development of the disease has shed new light on the causative mechanisms that may be behind this condition. Familial clustering of peripartum cardiomyopathy has been identified; however, on screening other family members and with further genetic testing, this clustering may represent a subset of undiagnosed familial dilated cardiomyopathy. TNF alpha, and other proinflammatory cytokines have been shown to be elevated in a large number of peripartum cardiomyopathy cases and similarly some studies have suggested a role for autoantibodies against normal human cardiac tissues proteins and further research is required in this area [92]. Higher levels of CRP, Fas/Apo-1, TNF alpha and IL-6 have been demonstrated in some population groups with peripartum cardiomyopathy and have implicated a role for inflammatory mediator in the disease process [93, 94]. The use of immunoglobulin and antitumour necrosis factor agents as therapy for peripartum cardiomyopathy has been trialled based on these observations in several smaller pilot studies [95, 96]. Evidence implicating myocarditis as a causative factor is varied however may suggest the presence of myocarditis in 7.8–8.8% of cases [97]. The role of an altered form of prolactin in the pathophysiology of peripartum cardiomyopathy has been explored of late in animal models. Mice with cardiac tissue-specific STAT3 knockout have shown an increased cleavage of prolactin (a pituitary hormone released cyclically in varying degrees in the pregnant state) mediated by cathespsin D to its proapoptotic and antiangiogenic form, 16 kDa prolactin and have subsequently demonstrated the development of peripartum cardiomyopathy [98]. In light of this research, further preliminary studies have taken place on the use of bromocriptine as a therapy for women developing peripartum cardiomyopathy this small trials conducted thus far have shown a mortality benefit [99]. Peripartum cardiomyopathy, although rare, is an important entity affecting the pregnant woman, with significant morbidity and mortality consequences. Recent research into the pathophysiology behind the disease may allow for further subclassification of this disease, and hence earlier diagnosis, and new novel therapies in its treatment.

### 2.4. Arrhythmogenic Right Ventricular Dysplasia (ARVD)

ARVD is a heart muscle disease which, pathologically, consists of progressive fibrofatty replacement of the right ventricular musculature which may or may not involve the left ventricle. It predisposes towards malignant arrhythmias originating from the right ventricle and is a major cause of sudden death in young athletes [100]. Major and minor criteria of ARVD diagnosis have been compiled, and the diagnosis can be made if there are two major, one major and one minor or four minor criteria present [101]. Diagnosis and risk stratification are extremely important as there are proven life saving interventions which are available to the clinician [102].

It is a familial disease in around 50% of cases and is usually transmitted in an autosomal dominant fashion [103]. The first gene, ARVD1, coding for a desmosome protein, was discovered in 1994 [42], and since then multiple causative genes relating to the desmosome have been discovered, indicating that ARVD is a disease of the desmosome [55, 57, 104–107] (Table 4).

Genetics is obviously important as it adds certainty to the diagnosis, but given the incomplete penetrance of the disease the established diagnostic criteria are essential.

## 3. Discussion

The 2009 Heart Failure Society of America (HFSA) genetic evaluation of cardiomyopathy practice guideline and 2005 American College of Cardiology/American Heart Association (ACC/AHA) HF guidelines include recommendations with regard to genetic counselling and genetic testing in patients and families with certain cardiomyopathies. As far as treatments are concerned, gene therapy is still quite young and transferring concepts from animal models to human therapies is yet to be seen. Therapeutic interventions of the future are likely to focus on the signaling events from abnormal gene to protein and finally clinical phenotype, and the modification of the genetic and environmental factors mediating this process [108].

As sudden cardiac death is a possible first presenting complaints for patients with dilated cardiomyopathy especially those with *LMNA* gene defects (where penetrance rates are noted to be very high over 30 years and associated with high rates of sudden death) and in particular *SCN5A* defects, early implantation of ICD may be considered in these populations, especially in the setting of family history of sudden cardiac death or implantable cardiac defibrillator usage [109].

Gene therapy in animal models of heart failure aimed at improving sarcoplasmic calcium transport has been investigated with therapeutic promise and may lead to further application in human model of restrictive cardiomyopathy [110].

In patients with ARVC, genotyping and early ICD implantation as primary prevention may be indicated in patients with Naxos disease and recessive forms of ARVC [111].

### 3.1. The Impact of Genetic and Genomic Approaches on Current and Future Clinical Application

Screening of family members of patients with dilated cardiomyopathy, hypertrophic cardiomyopathy, and arrhythmogenic right ventricular is recommended as family members are frequently asymptomatic and disease progression is often quite short and although asymptomatic early noninvasive investigations may prove abnormal [112, 113].

Due to the commonality of autosomal dominant inheritance of hypertrophic cardiomyopathy and the high degree of penetrance associated with many of the gene mutations, it is recommended that first-degree relatives are regularly screened for inheritance of the disease [114]. With the likely increase in amount of genetic testing the impact on family members of patients with an inheritable disease is likely to be affected significantly, particularly with many defects identified showing varying degrees of expression and penetrance. The role of genetic counselling will become more and more important as further genetic variants are identified with unknown pathological and prognostic significance.

For example, patients with gene mutations of desmosomal components (those most commonly seen in ARVC), penetrance is low and there is commonly age related variability in expression and therefore, early identification holds an unknown prognostic significance for patients in question [115, 116].

## 4. Conclusion

Classification systems in all branches of science are designed to allow categorisation within a consistent framework thereby imparting a degree of homogeneity to satisfy researchers and clinicians alike. Over the years, many classification systems have been put forward for cardiomyopathy based on origin, structural abnormality, functional status, and etiology. Not surprisingly, this has failed to some degree. From a purely functional viewpoint, cardiomyopathy is not a static condition but can move from one functional group to another due to cardiac remodelling. Similarly, using etiology has limitations given that similar genotypes can express different phenotypes depending on where the disease is in its natural history. Despite these shortcomings, a genetic diagnosis does offer some definite advantages. Karibe et al. reported a novel tropomyosin mutation that was associated with a mild phenotype but had a poor prognosis when contrasted to other mutations in the gene (13 deaths in 26 affected family members) [117]. Genetic testing in such cases allows identification of patients which would benefit from primary ICD implantation as well as a definitive diagnosis in conjunction with traditional methods. Similarly, patients with mutations in the gene for cardiac myosin-binding protein C can have a favourable clinical course due to the fact that the cardiomyopathy may not be expressed until later in life. Therefore, prolonged lifetime screening for family members who do not have the mutation can be avoided by genetic testing within these individuals [118]. There has been some debate on the value of genetic testing, with the debate focusing on the ability of genetic testing to accurately predict clinical course [119]; however, genetic testing allows clinicians to move beyond unexplained ventricular abnormalities and definitively identify not only who has the disease but what the cause is and what are the likely outcomes. Changing the natural history of a disease starts with accurate diagnosis.

## Figures and Tables

**Table 1:** Summary of AHA 2006 classification [1].

Primary cardiomyopathies	Secondary cardiomyopathies
Genetic (hypertrophic cardiomyopathy; conduction abnormalities: prolonged QT syndrome; Brugada syndrome)	Infiltrative (amyloidosis and Gaucher disease)
Mixed (dilated cardiomyopathy; restrictive cardiomyopathy)	Storage (haemochromatosis and Fabry's disease)
Acquired (inflammatory myocarditis, peripartum, stress cardiomyopathy—“broken heart syndrome” or tako-tsubo)	Toxicity (drugs, alcohol, heavy metals, and chemicals/chemotherapy)
	Inflammatory (sarcoidosis) endocrine (diabetes mellitus; thyroid disorders; hyperparathyroidism), cardiofacial (Noonan syndrome, lentiginosis) neuromuscular/neurological, nutritional deficiencies, and autoimmune and collagen disorders

**Table 2:** Genes associated with hypertrophic cardiomyopathy.

Gene	Protein	Function	Reference
*β*-MHC	*β*-Myosin heavy chain	Sarcomere protein	[5]
*α*-MHC	*α*-Myosin heavy chain	Sarcomere protein	[6, 7]
cMYBPC	Cardiac myosin-binding protein C	Sarcomere protein	[8, 9]
cTnI	Cardiac troponin I	Sarcomere protein	[10]
cTnT	Cardiac troponin T	Sarcomere protein	[11]
cTnC	Cardiac troponin C	Sarcomere protein	[12]
*α*-TM	*α*-Tropomyosin	Sarcomere protein	[11]
MLC-1	Myosin essential light chain	Sarcomere protein	[13]
MLC-2	Myosin regulatory light chain	Sarcomere protein	[7]
ACTC	Actin	Sarcomere protein	[14]
TTN	Titin	Sarcomere protein	[15, 16]

Metabolic phenocopies			
PRKAG2	AMP kinase		[17]
LAMP2	Lysosome membrane protein		[18]

**Table 3:** Genes associated with dilated cardiomyopathy.

Gene	Protein	Function	Reference
Autosomal dominant			
ACTC	Cardiac actin	Sarcomere protein	[19]
DES	Desmin	Dystrophin-associated glycoprotein complex	[20]
SGCD	*δ*-Sarcoglycan	Dystrophin-associated glycoprotein complex	[21]
MYH7	*β*-Myosin heavy chain	Sarcomere protein	[22, 23]
TNNT2	Cardiac troponin T	Sarcomere protein	[22, 24, 25]
TPM1	*α*-Tropomyosin	Sarcomere protein	[26]
TTN	Titin	Sarcomere structure	[27]
VCL	Metavinculin	Intercalated discs	[28]
MYBPC	Myosin-binding protein C	Sarcomere protein	[23]
MLP/CSRP3	Muscle LIM protein	Z discs	[29]
ACTN2	*α*-Actinin-2	Sarcomere structure	[30]
MYH6	*α*-Myosin heavy chain	Sarcomere protein	[31]
ABCC	SUR2A	Cardiac K channel	[32]
LMNA	Lamin A/C	Nuclear membrane protein	[33]
PLN	Phospholamban	Sarcoplasmic reticulum Ca regulator	[34, 35]
ZASP/LBD3	Cypher	Cytoskeletal assembly	[36]

X linked			
DMD	Dystrophin	Dystrophin-associated glycoprotein complex	[37, 38]
TAZ/G4.5	Tafazzin		[39, 40]

Recessive			
TNNI3	Troponin I	Sarcomere protein	[41]

**Table 4:** Genes associated with ARVD.

Locus	Gene	Protein	Function	References
ARVD1	TGFB3	Transforming growth factor *β*3	Cell signalling	[42, 43]
ARVD2	RYR2	Ryanodine receptor 2	Sarcoplasmic reticulum calcium channel	[44, 45]
ARVD3	Not known	[46]
ARVD4	Not known	[47]
ARVD5	LAMR1	Extracellular matrix glycoprotein	Cell signalling, adhesion, and migration	[48, 49]
ARVD6	PTPLA	Protein-tyrosine phosphatase-like member A	Fatty acid synthesis	[50, 51]
ARVD7	DES; ZASP	Desmosomal protein; PDZ domain protein	Dystrophin-associated glycoprotein complex, andCytoskeletal assembly	[52, 53]
ARVD8	DSP	Desmoplakin	Anchoring of intermediate filaments	[53, 54]
ARVD9	PKP2	Plakophilin 2	Cell adhesion	[55, 56]
ARVD10	DSG2	Desmoglein 2	Calcium-binding transmembrane glycoprotein	[57, 58]
ARVD11	DSC2	Desmocollin 2	Calcium-dependent glycoprotein	[59, 60]
